# Research on Instance Segmentation Algorithm for Caged Chickens in Infrared Images Based on Improved Mask R-CNN

**DOI:** 10.3390/s25196237

**Published:** 2025-10-08

**Authors:** Youqing Chen, Hang Liu, Lun Wang, Chen Chen, Siyu Li, Binyuan Zhong, Jihui Qiao, Rong Ye, Tong Li

**Affiliations:** 1 College of Mechanical and Electrical Engineering, Yunnan Agricultural University, Kunming 650201, China; naniyafeng@163.com (Y.C.);; 2 The Key Laboratory for Crop Production and Smart Agriculture of Yunnan Province, Yunnan Agricultural University, Kunming 650201, China; 3 School of Big Data, Yunnan Agricultural University, Kunming 650201, China

**Keywords:** Mask R-CNN, caged chickens, instance segmentation, CBAM, AC-FPN

## Abstract

Infrared images of caged chickens can provide valuable insights into their health status. Accurately detecting and segmenting individual chickens in these images is essential for effective health monitoring in large-scale chicken farming. However, the presence of obstacles such as cages, feeders, and drinkers can obscure the chickens, while clustering and overlapping among them may further hinder segmentation accuracy. This study proposes a Mask R-CNN-based instance segmentation algorithm specifically designed for caged chickens in infrared images. The backbone network is enhanced by incorporating the CBAM within this algorithm, which is further combined with the AC-FPN architecture to improve the model’s ability to extract features. Experimental results demonstrate that the model achieves average AP and AR^10^ values of 78.66% and 85.80%, respectively, in object detection, as per the COCO performance metrics. In segmentation tasks, the model attains average AP and AR^10^ values of 73.94% and 80.42%, respectively, reflecting improvements of 32.91% and 17.78% over the original model. Notably, among all categories of chicken flocks, the ‘Chicken-many’ category achieved an impressive average segmentation accuracy of 98.51%, and the other categories also surpassed 93%. The proposed instance segmentation method for caged chickens in infrared images effectively facilitates the recognition and segmentation of chickens within the challenging imaging conditions typical of high-density caged environments, thereby contributing to enhanced production efficiency and the advancement of intelligent breeding management.

## 1. Introduction

According to relevant research, it is projected that by 2050, chicken consumption will increase by 2.3 times compared to the levels in 2010 [1]. In China, total poultry egg production in 2024 alone totaled 35.88 million tons [2]. In recent years, driven by growing consumer demand, the trend towards large-scale farming has accelerated, with three-dimensional cage farming emerging as the primary breeding model [3]. Compared to free-range farming, large-scale, high-density, three-dimensional layered cage farming enhances breeding efficiency in confined spaces, conserves land resources, reduces construction costs per unit of breeding density, and contributes to increased farmer incomes [4]. However, this farming method has inadvertently resulted in deteriorating living conditions for chickens and the cross-infection of related diseases. By implementing automated systems for continuous monitoring of chickens, farmers can easily detect behavioral and health abnormalities without increasing the demand for physical labor [5].

Identifying and segmenting chicken targets in images are prerequisites and foundational elements for developing an automated monitoring system. Since the advent of deep learning theory, it has undergone rapid development and is now widely applied across various industries. An increasing number of studies leverage deep learning technologies, particularly convolutional neural networks, to achieve the identification and segmentation of chickens in images. For instance, Lamping et al. (2022) proposed ChickenNet, a deep convolutional neural network capable of detecting and segmenting hens while providing an overall assessment of feather condition by calculating a feather condition score for each detected hen [6]. Ji Hengyi et al. (2024) focused on caged broiler breeders and proposed the YOLO-FSG model, which incorporates lightweight improvements to the YOLOv8n model for identifying the feeding behavior of chickens [7]. Pei Wei et al. (2025) employed a real-time detection method for identifying deceased chickens in caged systems, utilizing an improved version of YOLOv8n [8]. However, many studies have primarily achieved the detection and segmentation of chickens using visible light images. In actual poultry farms, environmental dust and inadequate lighting conditions significantly impede the acquisition of high-quality images, complicating the process of obtaining usable data.

Infrared imaging technology effectively captures images in low-light environments and can penetrate smoke, dust, and vegetation, making it widely applicable across various fields [9]. Utilizing infrared imaging sensor-based solutions to tackle challenges such as complex environmental backgrounds and avian obstructions enhances the accuracy and efficiency of monitoring [10]. Xiao Yang et al. (2024) employed infrared thermal imaging in their research on individual chicken image segmentation, achieving notable success in segmenting the entire body of chickens; however, they encountered limitations related to flock density, obstructions, and chicken behavior [11]. Mahtab Saeidifar et al. (2024) successfully segmented individual free-range laying hens in infrared thermal images [12]. Since infrared thermal imaging relies on capturing thermal radiation to create thermal images, it is highly susceptible to interference from objects that emit or reflect heat, such as warm feces, areas where chickens have rested, and surfaces that can reflect heat. These objects can create high-temperature zones in infrared thermal images within a short time, presenting challenges for the segmentation of chicken images in infrared thermal imaging.

Mask R-CNN is a multi-stage architecture for instance segmentation that adheres to the detect-then-segment paradigm [13]. To address the challenges of mutual occlusion and overlap among chickens in large-scale cage rearing conditions, as well as the segmentation difficulties caused by cages, drinkers, low light, and complex environments, this study proposes an instance segmentation method for caged chickens in infrared images based on Mask R-CNN model. The aim is to achieve accurate recognition and segmentation of caged chickens in infrared imagery.

The key contributions of this work include the following:

(1) This study utilizes a thermal imaging camera to collect data on caged chickens. Key frames are extracted using the inter-frame difference method, followed by data augmentation of the original dataset. Ultimately, a comprehensive dataset of caged chickens under infrared imaging is established.
(2) The native Mask R-CNN model was enhanced by reconstructing the backbone network using the attention mechanism module. Additionally, the original Feature Pyramid Network (FPN) structure was improved, resulting in the development of a novel high-precision instance segmentation framework.
(3) The improved Mask R-CNN model achieved AP, AR^M^, and AR^L^ performance metrics of 80.78%, 82.63%, and 86.26% in segmentation, respectively. Additionally, it achieved segmentation accuracies of over 93% for all four types of chicken flocks in the test set, with the highest reaching 98.51%. This model enables precise identification and segmentation of individual and group chickens in high-density caged environments.

The organizational structure of the subsequent content in the article is as follows: Section 2 introduces the relevant literature on instance segmentation, providing a comprehensive overview of existing methodologies and their implications. Section 3 details the datasets utilized and the methodological approaches employed in this research. Section 4 presents a summary and analysis of the experimental results, highlighting key findings. Section 5 discusses the significance of instance segmentation in the context of health monitoring for caged chickens, outlining the advantages of the enhanced model and its practical applicability. Finally, Section 6 concludes the article by summarizing the findings and emphasizing both the application value and limitations of the proposed model.

## 2. Related Work

Currently, numerous academic fields are engaged in research on instance segmentation. For instance, magnetic resonance imaging serves as the primary tool for detecting anatomical and functional abnormalities in intervertebral discs. Malinda Vania and Deukhee Lee (2021) developed an automated method for instance segmentation of intervertebral discs [14]. The CNN are widely utilized for the analysis of visual images and were first employed in the 1990s for the recognition of handwritten characters [15]. Ross Girshick et al. (2014) innovatively combined region proposals with CNNs, leading to the introduction of R-CNN [16]. The distinguishing feature of R-CNN is its ability to cluster an input image based on attributes such as color, size, and shape, thereby generating candidate bounding boxes. Subsequently, the features of these candidate boxes are computed using a large convolutional neural network, culminating in bounding box predictions. In 2015, Ross Girshick proposed Fast R-CNN, which enhances the process by performing a single forward pass of the CNN on the input image to derive the feature map for the entire image [17]. Ren et al. (2015) introduced Faster R-CNN, which allows the two architectures to share parameters in the initial layers while performing distinct tasks in the final layers, significantly reducing both training and testing times compared to R-CNN and Fast R-CNN [18]. Furthermore, He et al. (2017) proposed Mask R-CNN, which builds upon Faster R-CNN by incorporating an additional branch for predicting object masks, thereby achieving pixel-level detection results [19].

These models have been applied across various disciplinary fields. Cong et al. (2023) proposed an instance segmentation algorithm based on an improved Mask R-CNN to facilitate dynamic monitoring of the growth status of sweet peppers [20]. Meanwhile, Ming Yuan et al. (2025) introduced a novel network, GR R-CNN, to achieve precise segmentation of ships [21]. Li W et al. (2021) constructed a chicken image segmentation dataset and proposed an effective end-to-end framework for chicken image segmentation [22]. Chen Y et al. (2025) introduced a semantic segmentation method for chicken parts based on lightweight DeepLabv3+ to meet the demand for precise segmentation of different poultry body sizes [23]. While these models exhibit commendable segmentation effectiveness and performance within their respective domains, most are specifically designed for visible light image segmentation, resulting in a paucity of research focused on instance segmentation of infrared thermal imaging. The image data employed in these studies are characterized by high clarity, allowing for target segmentation objects to be distinctly distinguishable from the background, thereby facilitating easier segmentation. In contrast, infrared thermal imaging presents a challenge, as the flock of chickens often merges seamlessly with the background environment, complicating accurate segmentation. This study introduces enhancements to the Mask R-CNN model to improve its adaptability and achieve precise instance segmentation of caged chickens in infrared thermal images. Comparative experiments were conducted with widely recognized instance segmentation models, including YOLACT [24], YOLOv8 [25], YOLOv9 [26], YOLOv11 [27], and YOLOv12 [28]. YOLACT, as a pioneer in real-time instance segmentation, laid the groundwork for single-stage segmentation through its innovative prototype mask method. In contrast, YOLOv8 to YOLOv12 exemplify the ongoing evolution of the YOLO series in the realm of instance segmentation. The results of these comparative experiments substantiate the advantages of the model proposed in this study.

## 3. Materials and Methods

This section first describes the collection and processing of the infrared image dataset of caged chickens. Subsequently, it presents the overall structure of the enhanced Mask R-CNN model and discusses in detail the various evaluation metrics employed to assess the model’s performance.

### 3.1. Data Collection

**Figure 1:**
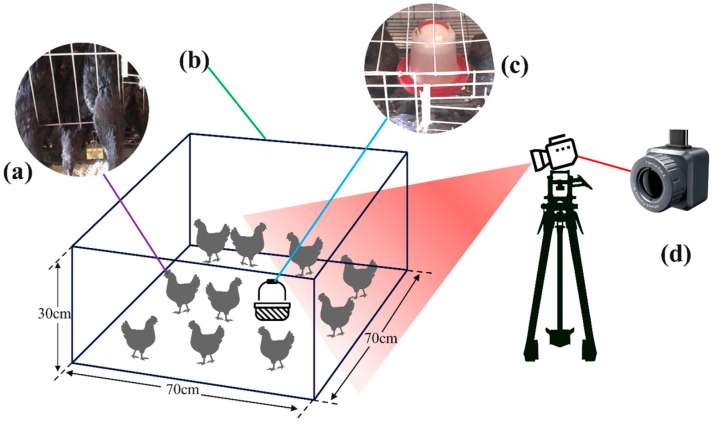
Schematic diagram of the collection system. (**a**) Chicken. (**b**) Chicken cage. (**c**) Water dispenser. (**d**) Thermal imaging camera.

The chicken breed utilized in this study is the Yanjin Silky fowl, sourced from the practical chicken farm of Yunnan Agricultural University in Yunnan Province, China. The China Iray Tianyan thermal imager served as the imaging equipment. Due to the three-dimensional stacked cage system in the chicken house, capturing images from above the cages was unfeasible. Consequently, the thermal imaging camera was inclined downward at a 45-degree angle relative to the horizontal plane and secured with a tripod for imaging. The schematic diagram of the collection system is presented in Figure 1. The data collection period spanned from 21 June to 11 July 2024. Chickens aged 8 to 10 weeks were selected, with 10 chickens from each of 10 distinct cages, resulting in a total of 100 chickens. Thermal imaging videos of the chickens were recorded daily from 9:00 to 23:00, maintaining a uniform video resolution of 720 × 540, in MP4 format, and a frame rate of 25 fps.

### 3.2. Data Preprocessing

**Figure 2:**
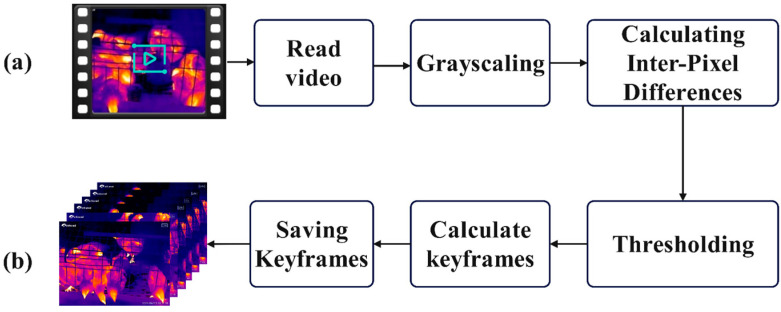
The processing flow of extracting key frames from video using the inter-frame difference method. (**a**) Video data. (**b**) Image data.

The thermal imaging video data of chickens cannot be directly utilized for model operation and requires further processing to convert it into image data suitable for model training. The video data consists of 25 frames per second, exhibiting minimal differences between adjacent frames. The motion states and distribution of the chicken flock in the processed image data must be unique and significantly distinct from other images. Therefore, it is essential to perform key frame extraction from the video to select appropriate images. To extract key frames from thermal imaging videos using the inter-frame difference method, the video images are first converted into grayscale images to reduce computational complexity. Subsequently, a difference operation is performed on the corresponding pixel points of two adjacent frames or multiple consecutive frames to calculate the absolute difference in grayscale values and obtain the average difference. The differenced images are then thresholded by setting a threshold T, marking the pixel points in the differenced images that exceed this threshold as key frames, as illustrated in Figure 2. Finally, 1548 original images of 720 × 540 pixels are retained, stored in PNG format, and all images are further scaled to 512 × 512 pixels.

### 3.3. Dataset Annotation and Augmentation

In this study, we utilize the image annotation tool Labelme (version 5.1.1) to systematically annotate the dataset. Caged chickens exhibit varying activity states throughout the day, typically resting during the noon and evening hours. Due to brief rest periods, attraction to feeders or water sources, and exposure to abnormal temperatures, chickens may lie down, press against one another, or closely adhere to each other. This behavior results in the formation of large contiguous and densely connected high-heat areas in infrared images. Consequently, this study categorizes the dispersion and aggregation states of chickens in infrared thermal imaging into four distinct classes, as illustrated in Table 1.

**Figure 3:**
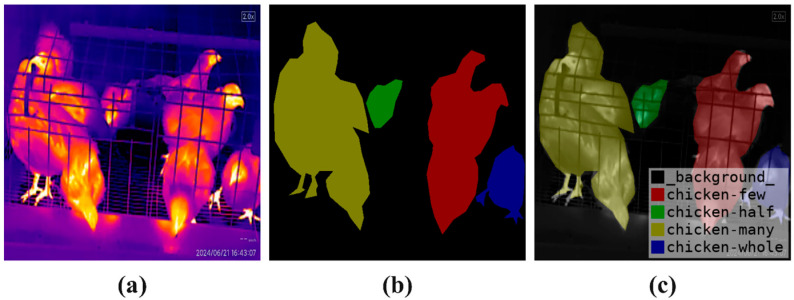
Image Annotation. (**a**) Original Image. (**b**) Mask Image. (**c**) Visualized Mask Image.

The annotation information generated after data annotation is stored in JSON format files, which are not directly usable for instance segmentation models. To address this limitation, the script provided by Labelme is utilized to convert the data into the Common Objects in Context (COCO) dataset format [29]. This conversion includes the original images, mask images, and visualized mask images, as illustrated in Figure 3.

**Figure 4:**
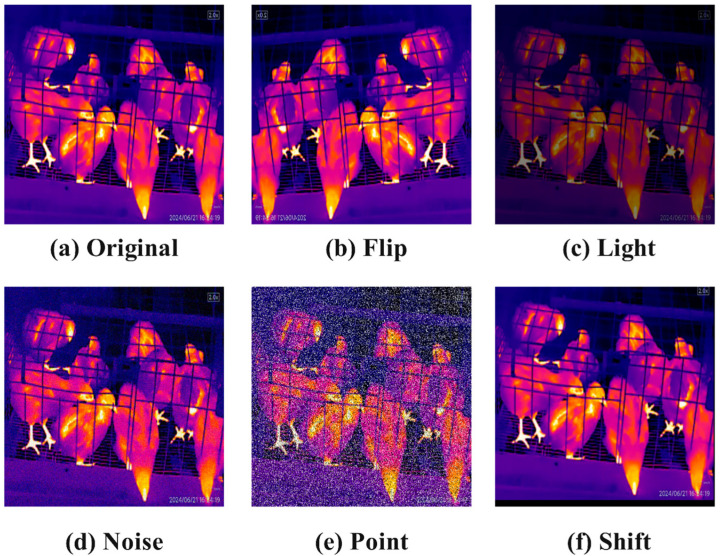
Effects of different enhancement methods. (**a**) Original image. (**b**) Image after mirror flipping. (**c**) Image after brightness adjustment. (**d**) Image after adding noise. (**e**) Image after randomly adding black and white dots. (**f**) Image after random translation.

Due to variations in lighting intensity, noise, and blurring during actual data collection, data augmentation techniques are employed to increase sample complexity and enhance the model’s generalization ability in complex scenarios. A total of 1548 images underwent mirror flipping, brightness adjustment, noise addition, random inclusion of black and white dots, and random shifting, resulting in an augmented dataset comprising 9288 images. We have quantified the quantities of each category: chicken-whole has 14,952, chicken-few has 8652, chicken-many has 7374, and chicken-half has 5454. The images enhanced through these different methods are illustrated in Figure 4.

### 3.4. Improved Mask R-CNN Model Architecture

**Figure 5:**
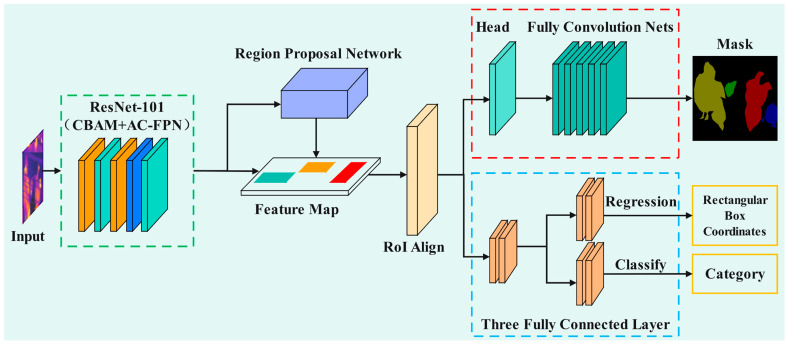
The structure of the improved Mask R-CNN model.

Mask R-CNN extracts regions and classifies bounding boxes through a dual-phase network. In the first stage, thermal imaging images of chickens are processed as input through a feature extraction backbone network to obtain feature maps. These feature maps are subsequently fed into the Region Proposal Network (RPN) to generate a series of candidate boxes [18]. In the second stage, each candidate box is passed through the RoIAlign layer for RoI pooling, resulting in feature maps of consistent size. These feature maps are then separately directed into the Mask branch (indicated by the red dashed box in Figure 5) and the Box and Class branches (indicated by the blue dashed box in Figure 5). The Mask branch is utilized to acquire segmentation information of objects within the images, while the Box and Class branches serve to obtain the location and category information of the objects. The overall structure of the improved Mask R-CNN model is illustrated in Figure 5. In Mask R-CNN, convolutional neural networks such as VGG16 [30] and ResNet [31] are typically employed for feature extraction; in this study, ResNet-101 is utilized. The input image size for the model is 512 × 512 pixels, and the model outputs chickens separated from the background, as well as categories classified based on varying states of dispersion and aggregation.

### 3.5. Integration of CBAM Attention Mechanism

**Figure 6:**
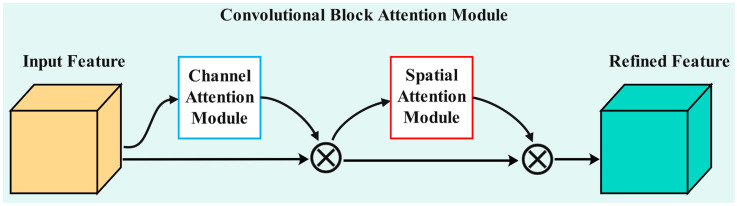
Structure diagram of the CBAM attention module.

Attention mechanisms are primarily applied across three dimensions: the channel dimension, the spatial dimension, and the hybrid dimension. The channel dimension focuses on feature channels, the spatial dimension emphasizes critical regions, and the hybrid dimension integrates both aspects. The CBAM is a lightweight attention module designed to enhance the performance of convolutional neural networks [32]. By combining channel attention and spatial attention, CBAM equips the model with more comprehensive and effective feature extraction capabilities. The core concept of the CBAM is to refine the input feature map in two stages: first, by learning the importance weights of different channels through the Channel Attention Module, and second, by determining the spatial importance distribution of the feature map through the Spatial Attention Module, as illustrated in Figure 6. Although CBAM introduces additional computational load to the model, its design has taken computational efficiency into account. Through global pooling and simple convolutional operations, CBAM can achieve performance improvements while maintaining a relatively low additional computational cost. In this study, CBAM was employed to enhance the ResNet-101 backbone network by integrating CBAMs between each residual block, thereby improving the Mask R-CNN model’s ability to focus on both channel and spatial dimensions when processing infrared images of caged chickens.

The output of the Channel Attention Module, *Mc(F)*, can be computed using the following formula:

(1) Mc(F)=σ(MLP(AvgPool(F))+MLP(MaxPool(F)))

In this study, we denote the input feature map as *F*. The operations referred to as *AvgPool* and *MaxPool* correspond to the global average pooling and max pooling operations, respectively. Furthermore, *MLP* stands for multilayer perceptron, while *σ* represents the Sigmoid activation function.

The output of the Spatial Attention Module, *Ms*(*F*), can be calculated using the following formula:

(2) $$Ms(F)=\sigma (f^{7\times 7}[AvgPool(F);MaxPool(F)]))$$

In the formula, $f^{7\times 7}$ represents a 7 × 7 convolution operation, and [*AvgPool*(*F*); *MaxPool*(*F*)] denotes the concatenation of the average pooling and max pooling results along the channel axis.

In the backbone network of the MaskRCNN model utilized in this study, which is constructed upon ResNet101, the placement of the Channel Attention Module and the Spatial Attention Module within the CBAM attention architecture adheres to a clear and consistent pattern. Both modules are embedded at the conclusion of each Bottleneck layer in ResNet101, specifically targeting the layers that follow each of the four primary feature extraction layers: layer1, layer2, layer3, and layer4. The Channel Attention Module is positioned first, immediately followed by the Spatial Attention Module. All Bottleneck layers across these four levels integrate both the Channel Attention Module and the Spatial Attention Module.

### 3.6. Improvement of the AC-FPN Module

FPN [33] serves as a fundamental module within the Mask R-CNN architecture, bridging the backbone network with subsequent tasks such as the RPN and RoIAlign. By incorporating a top-down pathway, FPN facilitates the generation of feature maps at various scales, each retaining robust semantic information. Nevertheless, its receptive field remains considerably smaller than the input data size. Feature maps associated with different receptive fields are combined solely through element-wise addition in the top-down pathway. This approach presents an inherent contradiction, as FPN aims to maintain high-resolution inputs while also striving for an expansive receptive field. Furthermore, there is a notable deficiency in effective communication between layers that operate at different receptive fields.

**Figure 7:**
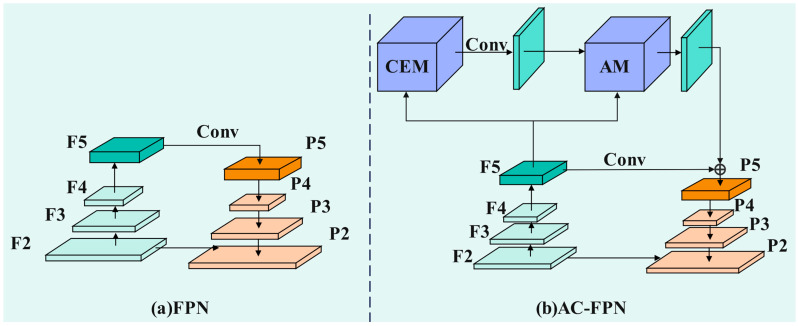
FPN and AC-FPN share the same fundamental structure, with the difference being that AC-FPN is equipped with CEM and AMs.

AC-FPN [34] enhances the standard FPN by incorporating two additional modules: the Context Extraction Module (CEM) and the Attention Guidance Module (AM). The CEM explores extensive contextual information from multiple receptive fields, expands the receptive field without sacrificing resolution, and fuses multi-scale contextual information. The AM adaptively captures salient dependencies of objects using an attention mechanism. By addressing the aforementioned issues of the FPN through these two modules, AC-FPN is utilized in this study to replace the original FPN. The structural comparison between FPN and AC-FPN is illustrated in Figure 7. After extracting multi-level features through the backbone network, the high-level features are first fed into the CEM. Deformable convolutions are introduced to enhance geometric modeling capabilities, ultimately fusing the original features with multi-scale context to generate enhanced features. Subsequently, these enhanced features enter the AM, where contextual attention computes global semantic associations of the feature maps through a self-attention mechanism to suppress redundant background interference, and content attention utilizes shallow features F5 to generate spatial weights, correcting positional offsets caused by deformable convolutions. Finally, the attention features output by the AM are fused with the lateral connection features of the traditional FPN, producing the optimized feature pyramid P2–P5, which is then used by the detection head to complete classification and regression tasks.

The F5 feature serves as the input to the CEM, which consists of two pathways: In the first pathway, a densely connected approach is employed where the F5 feature undergoes dilated convolutions with dilation parameters of 3, 6, 12, 18, and 24. This process extracts feature maps with varying receptive fields. To enhance the model’s ability to learn transformation-invariant features from the input data, deformable convolutions are incorporated at each connection. In the second pathway, to retain the coarse-grained information of the initial input, the F5 feature is subjected to upsampling. Ultimately, the outputs from both pathways are concatenated and processed through a 1 × 1 convolutional layer, which effectively fuses the coarse-grained and fine-grained features.

The AM consists of two parts: the Context Attention Module (CxAM) and the Content Attention Module (CnAM). The CxAM automatically focuses on more relevant sub-regions. For the given salient feature map $F\in R^{C*H*W}$. The convolutional layers $W_{q}$ and $W_{k}$ are used to transform it into a latent space, as described by the following formula:

(3) $$Q=W_{q}^{T}F\;\mathrm{and}\;K=W_{k}^{T}F$$

Then convert *Q* and *K* into vectors and process them further.

The CxAM transforms *F* using the following equation and obtains the correlation matrix:

(4) $$P=W_{p}^{T}F_{5}\;\mathrm{and}\;Z=W_{z}^{T}F_{5}$$

Then convert *P* and *Z* into vectors and process them further.

### 3.7. Evaluation Metrics

The dataset utilized in this study is structured in accordance with the standard COCO dataset; therefore, the COCO metrics are employed as evaluation criteria [29]. Given the significant proportion of chickens observed in infrared images, and considering the varying proportions they occupy based on their dispersion and aggregation levels, eight relevant metrics from the COCO metric system—namely, AP, AP^50^, AP^75^, AP^M^, AP^L^, AR^10^, AR^M^, and AR^L^—are selected for assessing model performance.

IoU represents the ratio of the overlapping area to the union area between the predicted box and the ground truth box. AP denotes average precision at 0.5 ≤ IoU ≤ 0.95, AP^50^ denotes average precision at IoU = 0.5, AP^75^ denotes average precision at IoU = 0.75, AP^M^ denotes average precision for medium objects (with area between 32^2^ and 96^2^ pixels) at 0.5 ≤ IoU ≤ 0.95, AP^L^ denotes average precision for large objects (with area greater than 96^2^ pixels) at 0.5 ≤ IoU ≤ 0.95, AR^10^ denotes average recall for all categories and all object sizes with a maximum of 10 detections per image at 0.5 ≤ IoU ≤ 0.95, AR^M^ denotes average recall for medium objects with a maximum of 100 detections per image at 0.5 ≤ IoU ≤ 0.95, AR^L^ denotes average recall for large objects with a maximum of 100 detections per image at 0.5 ≤ IoU ≤ 0.95.

## 4. Results

This section presents the results of all experiments conducted during the research, detailing the experimental environment and platform. It includes tests and ablation studies on the enhanced model, examines the impact of various backbone networks and FPN structures on the improved model, and conducts comparative experiments with other prevalent instance segmentation models.

### 4.1. Model Experimental Conditions

#### 4.1.1. Experimental Platform

The experiments in this study were conducted on the Windows 11 operating system, utilizing an Intel 12th Gen Core i9-12900KF CPU running at 3.20 GHz, accompanied by 64.0 GB of RAM (GIGA-BYTE TECHNOLOGY Co., Ltd., Taiwan, China). The GPU employed was an NVIDIA GeForce RTX 4090 with 24 GB of VRAM, utilizing CUDA version 12.8. The software was developed based on the PyTorch (PyTorch 2.5.0) deep learning framework and Python, with the development environment configured using PyCharm 2023 and Anaconda 3. PyCharm facilitates more effective debugging of project code and execution, while Anaconda handles the construction of the runtime environment and management of toolkits.

#### 4.1.2. Training Parameter Settings

Before training the dataset, it is randomly divided into training, validation, and test sets in a 7:2:1 ratio using an algorithm. The batch size for model training is set to the default value of 4, the initial learning rate is 0.004, and the number of training epochs is set to 70.

### 4.2. Testing of the Improved Mask R-CNN Model

The improved Mask R-CNN model was trained and tested over multiple iterations using both non-augmented and augmented datasets. Utilizing the non-augmented dataset, the model metrics stabilized after 70 training rounds. In terms of object detection, the enhanced Mask R-CNN model achieved average performance metrics of 78.66%, 91.32%, and 85.80% for AP, AP^50^, and AR^10^, respectively, as well as 78.40% and 86.64% for AR^M^ and AR^L^. For segmentation, the average performance metrics were 73.94%, 90.95%, and 80.41% for AP, AP^50^, and AR^10^, respectively, along with 70.70% and 81.67% for AR^M^ and AR^L^.

Training with the enhanced dataset, the improved Mask R-CNN model demonstrates superior performance in both object detection and segmentation compared to the model trained on the unenhanced dataset. Specifically, the average values of the object detection performance metrics, AP and AR^L^, reach 87.31% and 92.48%, respectively, indicating improvements of 8.65% and 5.84%. Similarly, the average values of the segmentation performance metrics, AP and AR^L^, attain 80.78% and 86.26%, respectively, reflecting enhancements of 6.84% and 4.59%. The experimental results of the enhanced Mask R-CNN model are summarized in Table 2.

**Figure 8:**
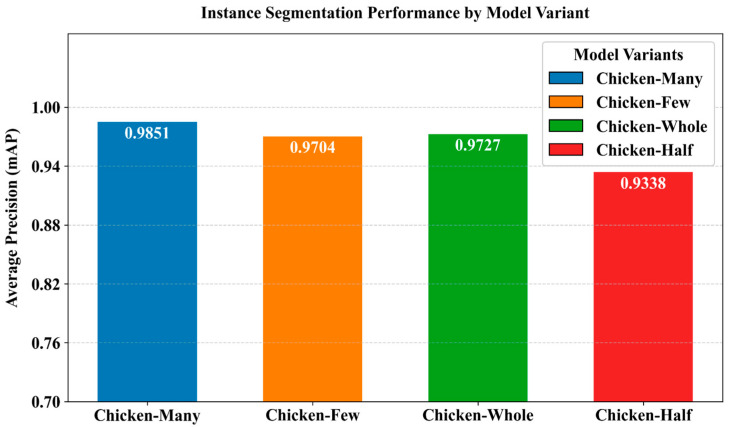
Average segmentation accuracy results across different categories.

After training the improved Mask R-CNN model, it was evaluated for its performance in image segmentation. Figure 8 illustrates the average segmentation accuracy results of the model across four categories. The model achieved average segmentation accuracies of 98.51%, 97.04%, 97.27%, and 93.38% for the categories Chicken-many, Chicken-few, Chicken-whole, and Chicken-half, respectively. The lower segmentation accuracy for the Chicken-half category can be attributed to the natural scarcity of half chickens, which is often caused by occlusion. The experimental results indicate that the model is capable of accurately segmenting chickens in infrared images.

### 4.3. Testing with Different Backbone Networks

To evaluate the performance of ResNet-101 as the backbone network for feature extraction in the instance segmentation of chickens in infrared images, we selected several backbone networks for the Mask R-CNN model to conduct performance testing: ResNet-50, DenseNet-121 [35], MobileNet [36], ConvNeXt [37,38], and ResNet-101. The experiments were carried out under consistent conditions to ensure a fair comparison, and the experimental results are presented in Table 3.

The results indicate that ResNet-101 outperforms all other architectures across most metrics, achieving AP and APL scores of 42.24% and 69.15%, respectively. This performance surpasses the corresponding parameters of the other four backbone network architectures. While ResNet-50 marginally exceeds ResNet-101 in terms of APL and AR10 metrics, it underperforms in all other metrics, particularly in ARM and ARL, where ResNet-101 demonstrates a significant advantage. In the context of infrared imaging of caged chickens, the representation of chickens predominantly includes medium and large objects. ResNet-101, with its deeper network architecture, exhibits enhanced model capacity and feature representation capabilities, resulting in superior performance on medium and large objects. Experimental data further reveals that DenseNet-121, MobileNet, and ConvNeXt consistently lag behind ResNet-101 across all metrics. While these architectures demonstrate advantages in their respective application scenarios, they also exhibit limitations in meeting the current task requirements. DenseNet-121 facilitates feature reuse through dense connections; however, its narrow layer design may dilute high-level semantic features. The COCO task necessitates robust spatial localization capabilities, and the gradient dispersion effect associated with dense connections may weaken the discriminative power of deep features. MobileNet, as a lightweight network architecture, reduces computational costs but at the expense of diminished feature extraction capabilities. Although ConvNeXt performs slightly better, it still does not match the performance of ResNet-101. Experimental results indicate that Mask R-CNN, utilizing the ResNet-101 backbone network, is more effective in identifying and segmenting caged chickens in infrared images.

### 4.4. Different FPN Tests

**Figure 9:**
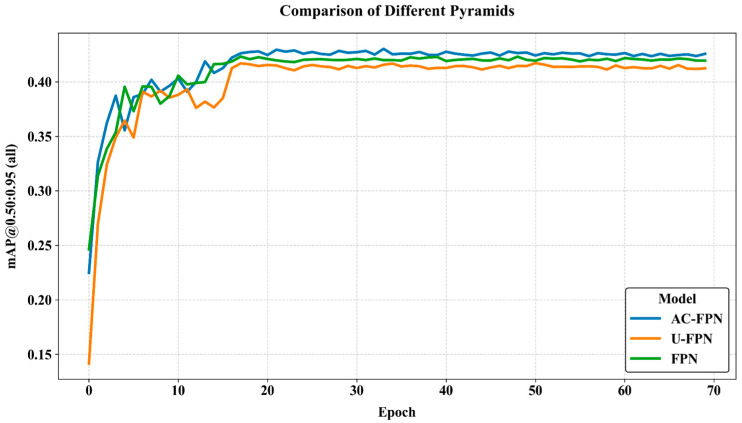
Experimental results of different FPN.

Tests were conducted on Mask R-CNN models equipped with FPN, U-FPN [39,40], and AC-FPN, respectively. In the COCO dataset, AP and mAP@0.5:0.95 are synonymous. Figure 9 illustrates the curves of the mAP@0.5:0.95 metrics for different FPNs as a function of the number of training epochs, visually demonstrating the performance of each FPN. The experimental results indicate that, after multiple training epochs, the mAP@0.5:0.95 metrics of all models tend to stabilize, with AC-FPN significantly outperforming the other two FPNs. These results further validate the effectiveness of AC-FPN in the instance segmentation task of caged chickens in infrared images.

### 4.5. Ablation Experiment

We designed four sets of experiments under identical conditions to verify the effectiveness of each module when acting independently. The results are summarized in Table 4. The experimental data indicate that the AP performance metrics of the Mask R-CNN models equipped with CBAM and AC-FPN, when evaluated separately, are both higher than those of the Mask R-CNN model without these two modules, and they outperform the original Mask R-CNN model across most metrics. This finding demonstrates that CBAM and AC-FPN can effectively enhance the model’s performance when utilized independently. Furthermore, the experimental data reveal that the model incorporating both CBAM and AC-FPN comprehensively outperforms the other three experimental groups, exhibiting significantly enhanced capabilities. The AP metric has improved significantly to 76.94%, marking a notable enhancement over the initial 42.32% achieved by the original Mask R-CNN model. The CBAM enhances the model’s feature extraction capability, enabling the extraction of more comprehensive and richer high-level features. The AC-FPN further improves the model’s feature representation and recognition ability, as indicated by the AR^10^ metric reaching 81.51%, demonstrating that the model can accurately capture the true locations of target objects even when detecting only a limited number of bounding boxes. The two modules exhibit strong synergistic effects, further enhancing the model’s feature extraction capabilities for medium and large objects. The performance metrics representing the model’s efficacy on medium and large targets, namely AP^M^, AP^L^, AR^M^, and AR^L^, reached 68.61%, 78.18%, 72.26%, and 82.69%, respectively.

**Figure 10:**
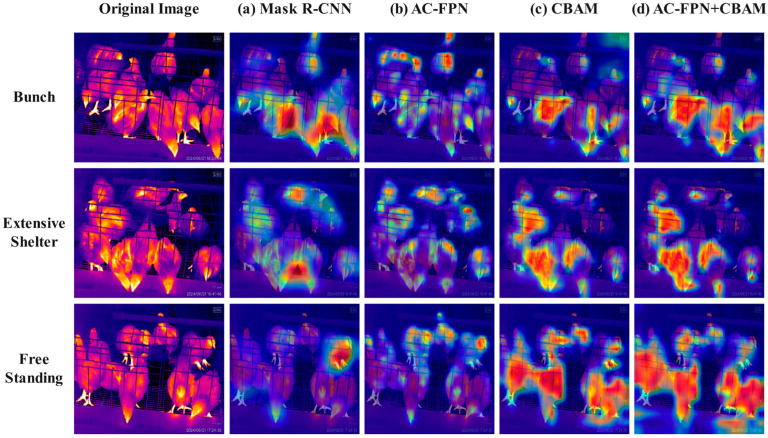
Heatmap. (**a**) Original Mask model. (**b**) Mask model with AC-FPN. (**c**) Mask model enhanced with CBAM attention mechanism. (**d**) Mask model incorporating both CBAM and AC-FPN.

In the subsequent study, the Grad-CAM plugin was utilized to generate heatmaps. The segmentation performance of four models was evaluated under three conditions: large flock aggregation, extensive occlusion, and scattered standing. The heatmaps of the segmentation results are depicted in Figure 10. Each colored point on the heatmap corresponds to a specific numerical value. The color gradient, transitioning from blue to red, indicates an increase in value from low to high. This gradient represents the progression of attention weights and activation levels, where red signifies high attention weights and activation levels. The original Mask R-CNN model with the ResNet-101 backbone exhibited activation in low-level feature areas, such as object edges, particularly in the floor and blank areas where chickens with specific temperatures had been located, failing to effectively focus on the chickens themselves. In contrast, the model with AC-FPN demonstrated high activation areas primarily on the chicken body parts, especially the head. The improved model exhibited a broader field of view and was better able to concentrate on the chicken body parts.

### 4.6. Comparison of Different Instance Segmentation Models

We conducted comparative experiments under the same experimental conditions. Once the models stabilized, the AP and AP^50^ metrics for object detection and segmentation were recorded and analyzed for each model. The experimental results are presented in Table 5. The findings indicate that, in terms of object detection performance, the AP metrics of other models hover around 49%, whereas the improved Mask R-CNN model achieves an AP metric of 82.46%, surpassing the YOLOv12 model by 31.11% (which records an AP of 51.35%). This suggests that the improved model outperforms the control group models, demonstrating its ability to accurately detect chickens in infrared images. In terms of segmentation performance, the improved Mask R-CNN model also achieves AP and AP^50^ metrics of 76.90% and 93.31%, respectively, while the relatively strong YOLOv12 model in the control group only records AP and AP^50^ metrics of 48.00% and 64.57% in segmentation.

**Figure 11:**
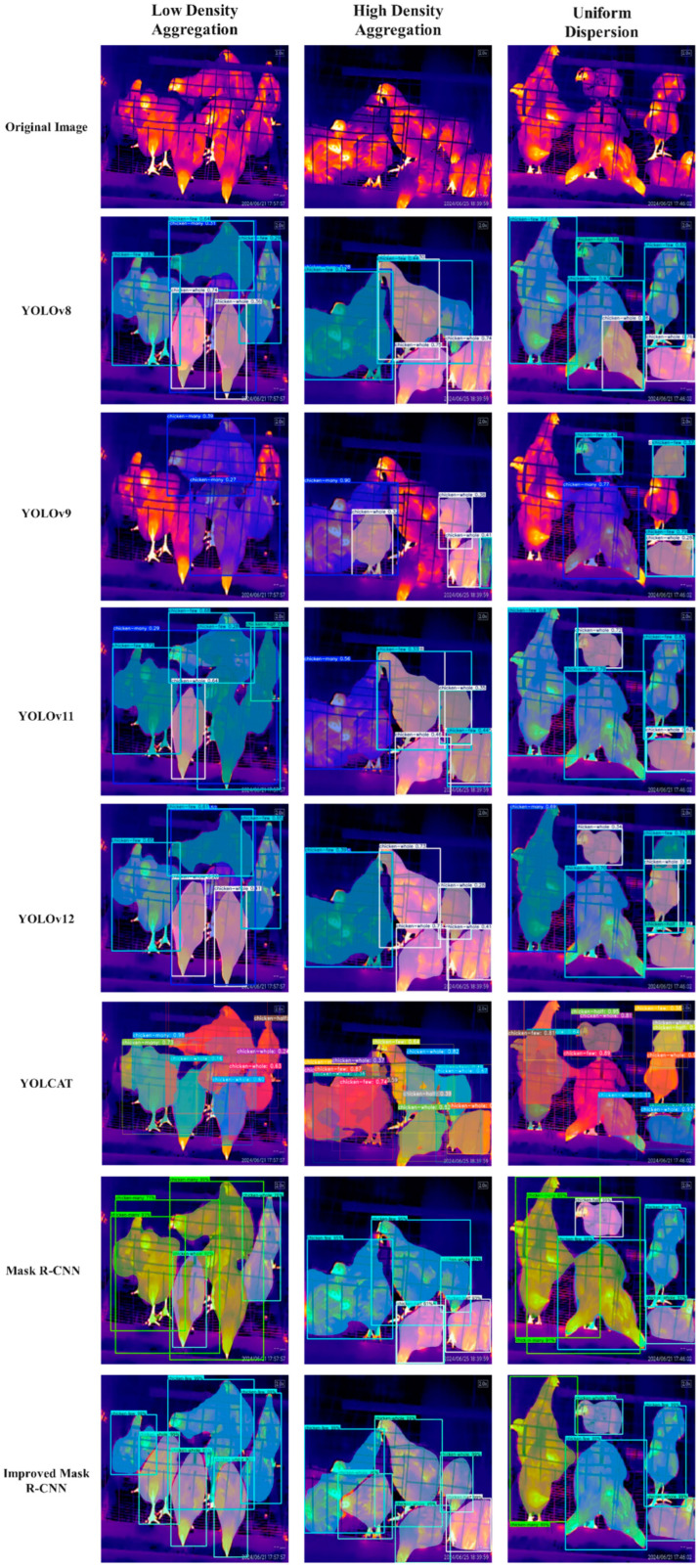
Segmentation results of different models.

Images representing low-density aggregation, high-density aggregation, and uniform dispersion were selected from the test set to evaluate each model, thereby providing an intuitive demonstration of the segmentation performance of different instance segmentation models, as illustrated in Figure 11. The test results indicate that both YOLOv8 and YOLOv9 demonstrate inadequate segmentation performance on the test images, failing to fully segment the chicken bodies. Specifically, detected individual chickens exhibit incomplete segmentation, with YOLOv9 revealing significant missed detections, while YOLOv8 suffers from issues of repeated detection. In contrast, the improved Mask R-CNN effectively detects all chickens in the images, completely separates the chickens from the background, and provides a more nuanced division of the chicken flock.

Although YOLOv11 and YOLOv12 represent the most advanced iterations of the YOLO series, the YOLO model functions as a single-stage detector. It processes the input image through a complex convolutional neural network (CNN) in a single pass, directly predicting the coordinates of bounding boxes, confidence scores, and class probabilities for each grid cell in the feature map. In contrast, the improved Mask R-CNN operates as a two-stage detector. In the first stage, the Region Proposal Network systematically scans the entire image, rapidly generating thousands of candidate regions that may contain objects, with these candidate boxes encompassing a broad range. In the second stage, fixed-size features are extracted from the backbone network’s feature map for each candidate region via the RoIAlign operation. These features are then processed through two parallel branches: the classification branch and the bounding box regression branch, which enhances segmentation precision compared to the YOLO series. Additionally, based on experimental results, the architecture of YOLCAT demonstrates suboptimal performance in the detection and segmentation tasks involving densely clustered caged chickens, yielding low metric scores; thus, it is deemed unsuitable for this application.

## 5. Discussion

Monitoring chicken flocks using infrared imaging represents a non-contact and stress-free approach that eliminates the necessity of capturing the chickens, thereby preventing additional stress responses within the flock. Such stress can adversely affect both health and productivity. In thermal imaging, the head and periocular regions of diseased chickens display abnormally high-temperature zones, creating a stark contrast with the surrounding healthy chickens. Conditions such as arthritis, footpad dermatitis, wing injuries, or localized infections can incite inflammatory responses, resulting in increased local blood flow and elevated temperatures. Image segmentation essential because the environmental temperature within the chicken coop is complex, with feed trays, water lines, chicken manure, and even walls potentially emitting thermal radiation. Without proper segmentation, these thermal interferences can significantly disrupt the measurement and analysis of the chickens’ body temperatures, leading to false alarms. Prolonged temperature monitoring of the isolated chickens can establish their normal body temperature fluctuation curve, thereby enabling the timely detection of abnormal body temperatures and facilitating necessary interventions.

This study proposes an enhanced Mask R-CNN model for the instance segmentation of caged chickens in infrared images. Test data indicate that the model achieves an average segmentation accuracy exceeding 93%, demonstrating its effectiveness in performing instance segmentation for caged chickens in infrared images. The improved Mask R-CNN achieves high-precision detection and pixel-level instance segmentation of core targets, ensuring greater accuracy and reduced miss rates in infrared images of caged chickens. Compared to the YOLO series and YOLCAT models, it demonstrates superior performance in precision and fine-grained analytical capabilities. This superiority arises from the inherent design of the Mask R-CNN model for instance segmentation, with its Region Proposal Network (RPN) serving as the cornerstone of its precision advantage. Consequently, this method can be employed to monitor the health status of chickens, thereby enhancing production efficiency, speed, and cost-effectiveness. It facilitates the identification of behavioral patterns associated with stress, fear, or discomfort in chickens. By understanding these behaviors, farmers can modify the environment to improve the living conditions of the chickens, ultimately leading to healthier and more productive poultry. For instance, it aids in determining optimal feeding times, adjusting temperature and lighting, and managing other environmental factors. Utilizing infrared cameras for detecting flocks of chickens can mitigate the limitations posed by visible light, which is affected by environmental conditions. Infrared images, based on thermal imaging principles, can assist in identifying early signs of disease in large-scale cage farming settings. This capability enables farmers to intervene promptly, preventing the spread of diseases and allowing for the identification and isolation of chickens that may carry or transmit infections.

## 6. Conclusions

In this study, we propose an enhanced Mask R-CNN instance segmentation model that optimizes both the backbone network and the FPN. This model demonstrates improved accuracy in identifying and segmenting caged chickens in infrared images. The primary conclusions are as follows:

(1) This study utilizes a thermal imaging camera to collect data on caged chickens. Key frames are extracted using the inter-frame difference method, followed by data augmentation of the original dataset. Ultimately, a comprehensive dataset of caged chickens under infrared imaging is established.
(2) The improved Mask R-CNN model achieved AP, AR^M^, and AR^L^ performance metrics of 80.78%, 82.63%, and 86.26% in segmentation, respectively. Additionally, it achieved segmentation accuracies of over 93% for all four types of chicken flocks in the test set, with the highest reaching 98.51%. This model enables precise identification and segmentation of individual and group chickens in high-density caged environments.
(3) The instance segmentation model has the disadvantage of poor real-time performance, and there is still room for improvement in inference speed and model size. Some common edge computing devices usually have limited performance, and the model needs to be further simplified to run well on such low-performance devices. At the same time, the datasets used in the research are still relatively scarce. In the future, it is necessary to further extend the research from caged chickens to free-range chickens and more breeds, and to achieve chicken monitoring in combination with visible light images.

The technique introduced in this paper provides a practical and viable resource for evaluating the health condition of caged chickens. This strategy facilitates precision farming and smart monitoring, improves productivity in the agricultural sector, encourages the use of artificial intelligence in farming, and establishes a foundation for more efficient health monitoring and management approaches.

## Figures and Tables

**Table 1:** Four annotation types.

Label Types	Annotation
Chicken-half	The obscured single chicken
Chicken-whole	Single chicken
Chicken-few	Small-scale gathering, 2–3 individuals
Chicken-many	Large-scale gatherings, four or more

**Table 2:** Experimental results of the improved Mask R-CNN.

Augmentation	Task	AP	AP^50^	AP^75^	AP^M^	AP^L^	AR^10^	AR^M^	ARL
×	Detection	0.7866	0.9132	0.8661	0.7705	0.7862	0.8580	0.7840	0.8664
√	Detection	0.8731	0.9511	0.9231	0.8877	0.8772	0.9155	0.8959	0.9248
×	Segmentation	0.7394	0.9095	0.8564	0.6517	0.7519	0.8041	0.7070	0.8167
√	Segmentation	0.8078	0.9492	0.9176	0.7774	0.8200	0.8520	0.8263	0.8626

Note: × denotes no data augmentation; √ denotes data augmentation.

**Table 3:** Experimental results of different backbone networks.

Backbone	AP	AP^50^	AP^75^	AP^M^	AP^L^	AR^10^	AR^M^	AR^L^
ResNet-50	0.4140	0.5935	0.4768	0.4244	0.4491	0.7041	0.5770	0.6500
DeseNet-121	0.2708	0.4746	0.2905	0.2606	0.2914	0.5844	0.5661	0.6097
MobilNet	0.2498	0.4336	0.2679	0.2144	0.2723	0.5833	0.4883	0.6098
ConvNeXt	0.3618	0.5867	0.4064	0.3637	0.3883	0.6191	0.5637	0.6483
ResNet-101	0.4224	0.5988	0.4777	0.4273	0.4456	0.6684	0.6223	0.6915

**Table 4:** Ablation experiment results.

CBAM	AC-FPN	AP	AP^50^	AP^75^	AP^M^	AP^L^	AR^10^	AR^M^	AR^L^
×	×	0.4232	0.6052	0.4787	0.4008	0.4506	0.6754	0.6086	0.7026
√	×	0.4243	0.6014	0.4841	0.4132	0.4522	0.6711	0.6084	0.6976
×	√	0.4303	0.6062	0.4850	0.4683	0.4541	0.6804	0.6657	0.7029
√	√	0.7694	0.9331	0.8897	0.6861	0.7818	0.8151	0.7226	0.8269

Note: × indicates the absence of the module; √ indicates the addition of the module.

**Table 5:** Experimental Results of Different Models.

Task	Indicator	YOLOv8	YOLOv9	YOLOv11	YOLOv12	YOLACT	Mask R-CNN	Improved Mask RCNN
Detection	AP	0.4962	0.4893	0.4832	0.5135	20.96	0.4384	0.8246
Detection	AP^50^	0.6295	0.6204	0.6078	0.6488	49.85	0.6150	0.9362
Segmentation	AP	0.4627	0.4581	0.4500	0.4800	28.89	0.4232	0.7690
Segmentation	AP^50^	0.6275	0.6231	0.6047	0.6457	50.43	0.6052	0.9331

## Data Availability

The data provided in this study can be obtained from the corresponding author upon request.
